# Impact of Endometrial Scratching on IVF/ICSI Outcomes: A Meta-Analysis

**DOI:** 10.3390/jcm15093340

**Published:** 2026-04-27

**Authors:** Rimantas Gricius, Kamilė Piesliakaitė, Ramunė Narutytė, Donatas Austys, Diana Ramašauskaitė

**Affiliations:** 1 Clinic of Obstetrics and Gynaecology, Institute of Clinical Medicine, Faculty of Medicine, Vilnius University, LT-03101 Vilnius, Lithuania; rimantas.gricius@mf.vu.lt (R.G.); ramune.narutyte@mf.stud.vu.lt (R.N.); diana.ramasauskaite@mf.vu.lt (D.R.); 2 Department of Public Health, Institute of Health Sciences, Faculty of Medicine, Vilnius University, LT-03101 Vilnius, Lithuania; donatas.austys@mf.vu.lt

**Keywords:** female infertility, repeated implantation failure, endometrial scratching, in vitro fertilization, intracytoplasmic sperm injection

## Abstract

**Background/Objectives**: Infertility affects about 17.5% of people globally, with higher rates in women. Despite advances in assisted reproductive technologies, success remains limited. Endometrial scratching (ES) is proposed to enhance implantation by altering cytokines and gene expression, but evidence is conflicting. **Methods**: A systematic search of the PubMed, Cochrane Library, and Scopus databases was conducted according to the Preferred Reporting Items for Systematic Reviews and Meta-Analyses (PRISMA) guidelines. Eight randomized controlled trials (RCTs) involving women undergoing in vitro fertilization (IVF) or intracytoplasmic sperm injection (ICSI), with ES performed in the luteal phase of the cycle preceding IVF or ICSI, were included. **Results**: The meta-analysis included 3210 patients and 1445 clinical pregnancies (754 in the ES group and 691 in the control group). In the ES group, a significantly higher clinical pregnancy rate (CPR) (RR 1.09, 95% CI 1.01–1.18, *p* = 0.02) was revealed. Pooled data from six studies reporting live birth rates (1268 births) showed a significant increase in the LBR after ES (RR 1.12, 95% CI 1.03–1.22, *p* = 0.01). The ongoing pregnancy rate (OPR) was also statistically significant in the ES group (RR 1.13, 95% CI 1.01–1.26, *p* = 0.03). **Conclusions**: This meta-analysis shows a small but statistically significant and consistent positive effect of ES on the LBR, CPR, and OPR in women undergoing IVF or ICSI, with a modest but measurable absolute benefit of approximately 4–5 additional events per 100 individuals. This procedure may particularly benefit patients with impaired endometrial receptivity, potentially enhancing reproductive outcomes while reducing the time and cost required to achieve a successful pregnancy. Further research is warranted to optimize its use and identify patients most likely to benefit.

## 1. Introduction

According to the World Health Organization, approximately 17.5% of the global population is affected by infertility, with a higher prevalence observed in females compared to males [1]. Similarly, the Centers for Disease Control and Prevention estimate that around 20% of married women in the United States experience difficulties conceiving [2]. Advances in infertility treatment have led to the increased accessibility and use of assisted reproductive technologies (ART). Despite advancements in ART, a 2023 publication by Adamson et al. reported that the global cumulative delivery rate after fresh or frozen embryo transfer stands at approximately 36.4% per aspiration [3]. ES has been proposed as a technique to enhance the likelihood of embryo implantation. Despite growing interest, there is an ongoing debate regarding the efficacy of ES in improving fertility outcomes, and the underlying biological mechanisms remain poorly understood. Some studies suggest that ES may enhance embryo implantation by increasing cytokine levels, such as IL-15, IL-17, and TNF-α, thereby maintaining the inflammatory state necessary for implantation [4,5]. Additionally, the protein osteopontin has been implicated in promoting angiogenesis and supporting fertility success [6]. Altered gene expression is also proposed as a mechanism through which ES may improve implantation rates. However, several concerns remain regarding its clinical application. The timing of ES appears critical, with the luteal phase being optimal for promoting implantation-related gene expression, whereas intervention during the proliferative phase may impair endometrial development [7]. Additionally, there is no clear consensus regarding the optimal frequency of ES prior to IVF cycles, with most studies performing a single procedure [8,9,10,11,12,13,14], while others perform ES twice [7]. Overall, the efficacy of ES remains controversial, with some experts advocating for its use, particularly in women with repeated implantation failure (RIF), and others cautioning against its widespread application, citing concerns over the limited clinical benefit, increased patient burden, and additional healthcare costs [15]. Several meta-analyses have evaluated the effectiveness of ES, with some suggesting a potential benefit [16,17,18]; however, the procedure remains a subject of ongoing debate. Further in-depth analyses are needed to more conclusively determine its efficacy and to identify specific patient populations that may derive the most benefit. This meta-analysis aimed to rigorously evaluate the effects of ES on key reproductive outcomes, including the LBR, CPR, and OPR, as well as the safety profile and other relevant pregnancy-related outcomes. The primary objective of this meta-analysis was to evaluate whether ES improves the CPR, LBR, and OPR in women undergoing IVF or ICSI.

## 2. Materials and Methods

### 2.1. Search Strategy and Study Selection

**Figure 1:**
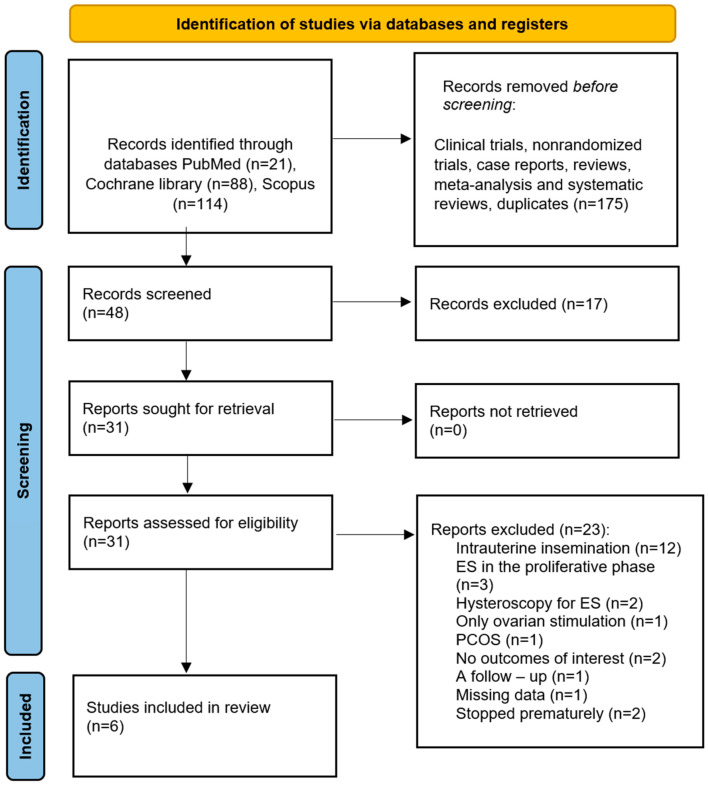
PRISMA flowchart.

The meta-analysis was prospectively registered in PROSPERO (CRD420251043665; registered 19 May 2025). The protocol prespecified the primary outcomes (LBR, CPR, and OPR), eligibility criteria (RCTs of luteal-phase ES prior to IVF/ICSI), and risk-of-bias assessment using the Cochrane RoB 2 tool, Bristol, United Kingdom. A predefined data synthesis plan included pooling of effect estimates for primary outcomes. Criteria for exclusion based on risk of bias, outlier handling, and detailed sensitivity analyses were not preregistered and these were conducted post hoc. The systematic search was conducted from 30 April 2025 to 30 May 2025 in the PubMed (*n* = 21), Scopus (*n* = 114), and Cochrane Library (*n* = 88) databases, yielding a total of 223 records. In PubMed, the filter “randomized controlled trial” was applied; in Scopus, “research articles” was used; and, in the Cochrane Library, “trials” was applied. The full search string used was ((endometrial scratching) AND ((IVF) OR (ICSI)) AND (((endometrial gene expression) OR (cytokines)) OR (((outcomes) OR (efficacy)) OR (feasibility)))), with equivalent adaptations across databases. The study selection process is shown in the PRISMA flowchart (Figure 1), with the complete PRISMA checklist provided in Supplementary Materials Table S5. AI-assisted language editing was conducted using GenAI (OpenAI, San Francisco, CA, USA). The authors reviewed and approved all text to ensure accuracy and integrity. Only RCTs were included; non-randomized trials, case reports, reviews, and non-English articles were excluded. Studies with patients having genital tract anomalies, recent uterine surgery, severe endometriosis, or severe male factor infertility were also excluded. Only RCTs with ES performed during the luteal phase and including participants with normal ovarian reserves were considered. Titles and abstracts were initially screened by two independent reviewers (K.P. and R.N.), followed by a full-text assessment to determine eligibility. A total of 31 full-text articles were assessed for eligibility. Of these, 12 [19,20,21,22,23,24,25,26,27,28,29,30] were excluded for using intrauterine insemination as the ART method; three [15,31,32] for performing ES in the proliferative phase; two [33,34] for using hysteroscopy for endometrial injury; one [35] for employing only ovarian stimulation as the ART method; one [36] for including only patients with PCOS; two [37,38] for not reporting the outcomes of interest; one [39] as it was a follow-up of an already included study; and one [40] due to missing data. Eight studies [7,8,9,10,11,12,13,14] involving 3500 participants (1750 in the ES group and 1750 in the control group) met the eligibility criteria. Six studies [7,8,9,11,12,13] comprising 3210 participants (1603 in the ES group and 1607 in the control group) were included in the primary meta-analysis after the exclusion of two high-risk-of-bias trials that reported outlying effect estimates, with sensitivity analyses incorporating all eight studies. The main characteristics of the included RCTs are demonstrated in Table 1.

### 2.2. Risk of Bias Assessment

All studies were assessed for risk of bias using the Cochrane Risk of Bias tool 2 (Supplementary Materials, Figure S1) by three independent reviewers (R.N., K.P., and D.A.), with discrepancies resolved through discussion. Domains assessed included randomization, intervention assignment, adherence, missing data, outcome measurement, and result selection. Judgments were categorized as “low risk”, “some concerns”, or “high risk”. Four RCTs [7,8,9,12] were rated as low risk, indicating high quality of evidence. Two RCTs were judged to be at a high risk of bias, primarily due to the early termination of the trials and relatively small sample sizes [10,14]. These trials also reported outlying risk ratios (RRs) and contributed to funnel plot asymmetry and were therefore excluded from the primary analysis and examined in sensitivity analyses. Mak et al., 2017 [13] raised some concerns in domain 2 for using a per-protocol analysis, although the findings were consistent with the intention-to-treat analysis and no patients were lost after embryo transfer; Metwally et al., 2021 [11] raised concerns in domain 3 due to over 20% missing data in both groups, despite attempting to address this through sensitivity analyses. Most studies on ES are not blinded [9,11,12,13,14], which could introduce bias by encouraging participants in the ES group to continue treatment when they otherwise would not [12]. However, since objective outcomes such as the CPR, LBR, and OPR are unlikely to be affected by the placebo effect, these studies were still classified as having a low risk of bias. Additionally, sham procedures may also cause bias through endometrial manipulation or damage [7]. The certainty of evidence of the included RCTs was assessed by the GRADE approach (Supplementary Materials, Figure S2).

### 2.3. Data Extraction and Statistical Analysis

Data extraction was carried out by two independent reviewers (K.P. and R.N.) and included study characteristics (such as author, year, country, and study period) and population characteristics (including mean patient age, mean BMI, mean duration of infertility, smoking status, cause and type of infertility, number of previous embryo transfers), as well as outcomes (CPR, LBR, and OPR), which were extracted and recorded in an Excel file. Baseline characteristics are summarized in Table 2. Table S1 (Supplementary Materials) provides the precise definitions of the outcomes measured in the included studies. The CPR was defined as the rate of pregnancies confirmed by ultrasound, showing a gestational sac with a detectable fetal heartbeat, 4 to 8 weeks after embryo transfer. The LBR was defined as the rate of deliveries with at least one live-born infant. The OPR was defined as the rate of pregnancies in which a detectable fetal heartbeat was present beyond 10 to 32 weeks of gestation. One study did not provide exact definitions for these outcomes [8]. All relevant data points were collected for each outcome. Table S2 (Supplementary Materials) summarizes the techniques, devices, and approaches to operator variability reported in the included RCTs. Devices were largely consistent across trials, with the Pipelle de Cornier most commonly used [7,8,9,10,14] and some studies referencing a generic Pipelle sampler [11], pipette [13], or endometrial biopsy catheter [12]. Only one study [10] reported that a single physician performed all procedures, while another [12] noted three operators without detailing standardization. All remaining studies provided no information on the number of operators or their training. Statistical analysis was conducted using IBM SPSS Statistics version 31.0. The CPR, OPR, and LBR were extracted from each study to calculate individual and combined RRs and confidence intervals (CI), which were then presented in several forest plots to assess and compare the effectiveness of ES in achieving these outcomes. A meta-analysis of binary outcomes was performed using the RR as the primary effect measure. Individual study effects were calculated on a logarithmic scale. Cohran’s Q test and I^2^, H^2^, and τ^2^ tests were performed to evaluate the heterogeneity of the results. A random-effects meta-analysis using the restricted maximum likelihood (REML) method was performed to obtain pooled estimates. Given the low-to-moderate between-study heterogeneity and the stability of the results in the sensitivity analyses, no additional variance adjustments (e.g., Hartung–Knapp correction) were applied. Inverse-variance weighting was applied. CIs were constructed using normal approximation and subsequently exponentiated to obtain pooled RRs. With respect to the fact that all included studies reported at least one event in each comparison group, no continuity correction was applied. As a secondary analysis, absolute risk differences (ARDs) were pooled using the same inverse-variance framework, and the number needed to treat (NNT) was calculated as the reciprocal of the pooled risk difference. To identify potentially influential or outlying studies, leave-one-out sensitivity analyses were performed, and funnel plot asymmetry was assessed to explore potential publication bias.

### 2.4. Missing Data

Missing data on baseline characteristics such as smoking status, the number of previous failed embryo transfers, the cause of infertility, and the type of infertility are detailed in the notes of Table 2. Patients with missing data were included in the denominator when calculating percentages. As not all trials reported the exact number of failed embryo transfers, patients were categorized as either undergoing their first transfer or having had at least one prior failed transfer. Complete outcome data were missing for 273 of the 1801 patients (15.2%) randomized to the ES group and 212 of the 1800 patients (11.8%) randomized to the control group. The missing data were primarily due to patients not undergoing embryo transfer, failure to undergo ES, loss to follow-up, discontinuation, secondary exclusion, or other reasons. Nevertheless, as most studies—except Mak et al. (2017) [13] and Frantz et al. (2019) [14]—employed an intention-to-treat approach, these patients were retained in the overall analyses and outcome calculations. As these two studies excluded patients with missing data post-randomization from their baseline characteristic analyses, these patients were likewise excluded from the pooled baseline analysis (Table 2).

## 3. Results

### 3.1. Descriptive Analysis of Participants

The pooled baseline characteristics are shown in Table 2. The pooled baseline characteristics of the participants of six studies were included in the meta-analysis, with comparison to all eight included studies. Participants in the ES and control groups had similar mean ages (34.89 vs. 34.96) and BMIs (23.99 vs. 24.28). The mean duration of infertility was 44.53 months in the ES group and 46.38 months in the control group. Approximately 40% of participants had zero or one previous failed embryo transfer in both groups, whereas around 12% had two or more failed transfers. Most studies did not specify the cause of infertility; when reported, common causes included male factor, idiopathic, tubal, mixed, endometriosis, ovulatory, or not specified. When reported, primary infertility was the most common, affecting 18.2% of the ES group and 17.9% of the control group participants.

### 3.2. Ovarian Stimulation Protocols

The different ovarian stimulation protocols used in the included RCTs are specified in the Supplementary Materials (Table S3). Most included studies [7,8,10,11,12,14] used standard ovarian stimulation in fresh IVF/ICSI cycles. Frantz et al. [14] used recombinant FSH with either a long GnRH agonist or antagonist protocol; Nastri et al. [10] permitted three routine regimens (clomiphene + hMG + antagonist, rFSH + antagonist, or rFSH + long agonist); Gibreel et al. [7] used combined oral contraceptive pill pre-treatment followed by long agonist downregulation; and Olesen et al. [8] applied a standardized rFSH + antagonist protocol. The van Hoogenhuijze [12] and Metwally [11] trials were pragmatic and followed each clinic’s usual stimulation practices. Two studies did not involve ovarian stimulation: Mak et al. [13] evaluated natural-cycle FET based on spontaneous ovulation, and Rodriguez et al. [9] studied donor egg recipients whose endometrium was prepared with estradiol only. All fresh-cycle trials that reported protocols used routine progesterone luteal support. Nastri [10], Gibreel [7], and Olesen [8] specified micronized progesterone or vaginal progesterone gel, whereas Frantz [14], van Hoogenhuijze [12], and Metwally [11] relied on standard clinic-based progesterone support. In the natural-cycle FET study [13], luteal support was optional. Donor egg recipients in Rodriguez et al. [9] received estradiol followed by vaginal micronized progesterone until the pregnancy test. Although stimulation and luteal support differed somewhat across studies, these factors were not randomized and were similar between arms within each trial, and they are therefore unlikely to explain between-group differences in outcomes, while they may contribute modestly to between-study heterogeneity.

### 3.3. Clinical Pregnancy Rate, Live Birth Rate, and Ongoing Pregnancy Rate

**Figure 2:**
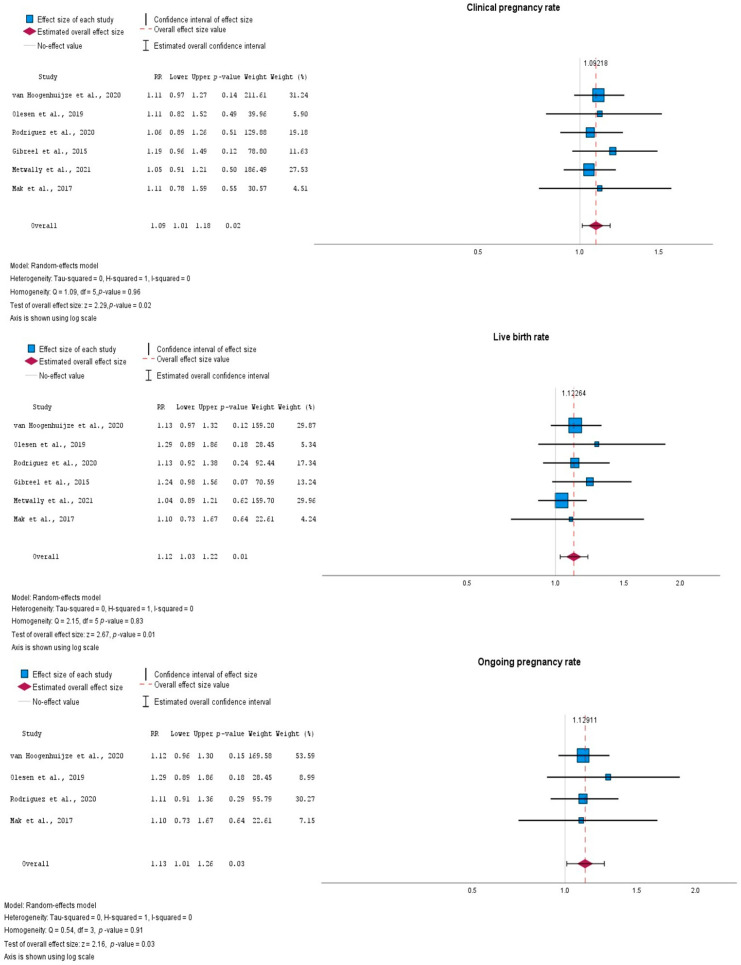
Forest plots, outcomes: clinical pregnancy rate, live birth rate, ongoing pregnancy rate [7,8,9,11,12,13].

In the main meta-analysis evaluating the CPR, six studies were included (Table 3). The pooled effect estimate was statistically significant (log RR = 0.088, SE = 0.038), corresponding to a pooled RR of 1.09 (95% CI 1.01–1.18; *p* = 0.022) (Figure 2). No between-study heterogeneity was observed (Q = 1.09, df = 5, *p* = 0.955; I^2^ = 0%; τ^2^ = 0). The pooled ARD was 0.04 (95% CI 0.01–0.07), yielding an estimated NNT of 25. When all eight eligible studies were included, the pooled effect remained statistically significant and of similar magnitude (RR = 1.10, 95% CI 1.02–1.18; *p* = 0.013). Between-study heterogeneity remained negligible (I^2^ = 0.2%; τ^2^ ≈ 0).

A total of six studies were included in the assessment of the LBR. The pooled effect estimate was statistically significant (log RR = 0.116, SE = 0.043), corresponding to a pooled RR of 1.12 (95% CI 1.03–1.22; *p* = 0.008) (Figure 2). No between-study heterogeneity was observed (Q = 2.15, df = 5, *p* = 0.829; I^2^ = 0%; τ^2^ = 0). The pooled ARD was 0.05 (95% CI 0.01–0.08), yielding an estimated NNT of 22.

When all seven eligible studies were included (Frantz et al., 2019 [14] did not measure this outcome), the pooled effect remained statistically significant and of similar magnitude (RR = 1.14, 95% CI 1.05–1.24; *p* = 0.002). Between-study heterogeneity remained negligible (I^2^ = 0.1%; τ^2^ ≈ 0).

In the main meta-analysis excluding two outlying studies and those with missing data about the OPR, four studies were included. The pooled effect estimate was statistically significant (log RR = 0.121, SE = 0.056), corresponding to a pooled RR of 1.13 (95% CI 1.01–1.26; *p* = 0.031) (Figure 2). No between-study heterogeneity was observed (Q = 0.54, df = 3, *p* = 0.910; I^2^ = 0%; τ^2^ = 0). The pooled effect was 0.05 (95% CI 0.01–0.10), yielding an estimated NNT of 20.

When all five eligible studies [8,11,12,13,14] were included (three studies [7,10,11] did not measure this outcome), the pooled effect did not remain statistically significant and corresponded to RR = 1.11, 95% CI 0.99–1.24; *p* = 0.062. Between-study heterogeneity remained negligible (I^2^ = 0.1%; τ^2^ ≈ 0).

### 3.4. Results for Fresh Embryo Transfers

When only studies describing procedures solely with fresh embryos were included [7,8,9,10,12,14], the pooled effect remained statistically significant and of similar magnitude for the CPR (RR = 1.11, 95% CI 1.02–1.22; *p* = 0.016) and LBR (RR = 1.19, 95% CI 1.07–1.32; *p* = 0.001). Between-study heterogeneity remained negligible (respectively, I^2^ = 0.1% and τ^2^ ≈ 0, I^2^ = 0.5% and τ^2^ ≈ 0). The pooled effect did not remain statistically significant for the OPR (RR = 1.11, 95% CI 0.99–1.24; *p* = 0.071; I^2^ = 0.1%; τ^2^ ≈ 0). Because of the missing data, OPR calculations did not include two studies [7,10].

### 3.5. Results in Patients with Repeated Implantation Failure

When only studies describing RIF were included [7,8,12,13], the pooled effect remained statistically significant and of similar magnitude for the CPR (RR = 1.13, 95% CI 1.02–1.25; *p* = 0.024) and LBR (RR = 1.17, 95% CI 1.04–1.32; *p* = 0.008). Between-study heterogeneity remained negligible (respectively, I^2^ = 0% and τ^2^ ≈ 0, I^2^ = 0% and τ^2^ ≈ 0). The pooled effect did not remain statistically significant for the OPR (RR = 1.14, 95% CI 0.99–1.30; *p* = 0.059; I^2^ = 0%; τ^2^ ≈ 0). Because of the missing data, OPR calculations did not include Gibreel et al., 2015 [7].

When only studies describing mixed groups (RIF/First) were included [9,10,14], substantial heterogeneity and non-negligible between-study variance were observed. None of the pooled effects remained statistically significant: CPR (RR = 1.08, 95% CI 0.66–1.76; *p* = 0.759; I^2^ = 81.5%; τ^2^ ≈ 0.15), LBR (RR = 1.37, 95% CI 0.86–2.17; *p* = 0.187; I^2^ = 69.6%; τ^2^ ≈ 0.08), OPR (RR = 0.93, 95% CI 0.57–1.52; *p* = 0.758; I^2^ = 63.3%; τ^2^ ≈ 0.09).

## 4. Discussion

### 4.1. Mechanism of Endometrial Scratching

The underlying mechanism by which ES may enhance conception rates remains unclear; however, several studies suggest that ES induces physiological and molecular changes in the endometrium. Research has shown alterations in gene expression, cytokine profiles, and immune cell activity following the procedure. A 2010 study reported elevated levels of cytokines such as growth-regulated protein alpha (GRO-α), interleukin-15 (IL-15), macrophage inflammatory protein-1β (MIP-1β), and tumor necrosis factor-alpha (TNF-α) in the ES group compared to controls [4]. Furthermore, increasing TNF-α levels directly correlated with increasing levels of MIP-1B, mucin 1 (MUC1), GRO-α, and IL-15. Higher levels of macrophages, dendritic cells, and osteopontin (OPN) were also detected, while no significant changes in vascular endothelial growth factor (VEGF) were observed [4]. TNF-α helps to maintain the inflammatory state of the endometrium required for trophoblast implantation [5]. It also activates IL-17, a cytokine that promotes gene expression that is critical for trophoblast migration and invasion [5]. Moreover, one study found significantly higher levels of OPN, an extracellular matrix protein involved in embryo implantation, in the endometria of women who had undergone successful IVF or ICSI procedures [6]. Furthermore, the same study showed that the OPN levels were higher in the luteal phase (when endometrial decidualization occurs) and it possibly activates VEGFA and VEGFR2 (angiogenesis factors). Angiogenesis is essential in providing the developing embryo with the necessary nutrients and oxygen. Disrupted expression of angiogenesis-related genes interferes with successful implantation and may lead to infertility [41]. Women with RIF exhibit lower levels of angiogenic factors in the endometrium [42]. In one study, changes in the expression of 84 angiogenesis-regulating genes were investigated after ES [43]. The results showed that 80% of these genes were downregulated and 20% upregulated. The expression of FGF1, VEGFA and IL-12A, and VEGFR1 and VEGFR2 was statistically significantly higher in the ES group than in the control group (*p* < 0.001), while the expression of IL-12B, IL-17F, COL18A, SERPINF1, TNF-α, and CXCL11 was significantly lower after the procedure [43]. FGF1 expression is decreased in the endometria of women experiencing RIF, which is important because this protein family contributes to the formation of blood vessels in the endometrium and to trophoblast implantation [43,44]. A randomized controlled trial using power Doppler imaging also demonstrated increased endometrial vascularization indices after ES, suggesting enhanced angiogenesis [10].

In women with unexplained RIF, ES performed during the proliferative phase led to the downregulation of innate (e.g., CCL2, TLR3, TLR4) and adaptive (e.g., CXCR3, IFNγ, IL-17A) immune genes, while upregulating anti-inflammatory markers such as IL-4 and CSF2. The overexpression of CXCR3 and IFNγ has been associated with Th1 activation and adverse pregnancy outcomes; thus, their reduction post-ES may be beneficial [32]. Another study investigating changes in gene expression after ES on days 11–13 and 20–24 of the cycle found that 183 genes were upregulated and 39 were downregulated after the procedure [45]. The most notable were increases in mRNA for uroplakin Ib (UPIb), adipose differentiation-related protein (ADFP), and mucin 1 (MUC1). In this study, for the first time, an increase in UPIb was observed. Although this protein family is localized in the urothelium, UPIb is the only protein of this family found in the secretory vesicles of endometrial glandular cells, which may indicate its involvement in endometrial glandular function [45]. In this study, as in the one mentioned above, an increase in MUC1 was observed after ES [4,45]. This is important for successful trophoblast implantation into the endometrium, as several previous studies have shown a statistically significant decrease in MUC1 levels in endometrial cells in women with RIF or spontaneous miscarriage [46,47].

### 4.2. Outcomes in Unselected Patients

Overall, our meta-analysis showed that ES was associated with modest but statistically significant improvements in the CPR and LBR, with consistent effect sizes across primary and sensitivity analyses. Subgroup analyses restricted to fresh embryo transfers and RIF populations yielded similar significant effects, whereas mixed populations demonstrated substantial heterogeneity and no significant benefit. Although the OPR showed a consistent direction of effect, statistical significance was not maintained across all analyses. Two RCTs [10,14] appeared as outliers in the funnel plots, inconsistent with the overall evidence pattern, and, after risk of bias evaluation, were judged to be at high risk of bias. Although the relative effect was modest, the corresponding ARD indicates a measurable population-level impact: from four to five additional events per 100 individuals. Importantly, heterogeneity was negligible in all main analyses, supporting the robustness of these findings. Although clinical, ongoing pregnancies, and live births were generally more frequent in the ES groups, the differences were not statistically significant in most individual studies [7,8,9,11,12]. However, the pooled analysis demonstrated a significant increase in the CPR, LBR, and OPR. Similar findings have been reported in other meta-analyses, with a significantly higher LBR and CPR observed following ES [16,48], while the results for the OPR are inconsistent [16,17]. One of the outlying studies involving women undergoing their first or second IVF cycle was prematurely terminated due to a non-significant trend toward a lower CPR in the ES group (23.5% vs. 35.9%; HR = 0.43, 95% CI: 0.18–1.02; *p* = 0.0568) [14]. However, this finding was not consistent with other studies, which generally reported a trend toward a higher CPR following ES [7,8,9,11,12,13]. Another study found no effect of ES after fresh embryo transfers in natural cycles but speculated that it may be more beneficial for an abnormally developing endometrium, as seen in stimulated cycles [13]. The second outlying study found a significant increase in the LBR (41.77% vs. 22.78%), CPR (49.37% vs. 29.11%), and implantation rate after ES. However, most participants had at least two prior failed embryo transfers, limiting the finding’s generalizability to all IVF patients [10]. The study also observed a significant increase in endometrial vascularization via three-dimensional power Doppler, offering insights into the mechanisms of ES [10]. No significant differences in the LBR, CPR, or miscarriage rate were observed when analyzed by embryo transfer stage (cleavage or blastocyst) or the time interval between ES and transfer [9]. A subgroup analysis suggested potential benefits for day-5 transfers or cycle programming with oral contraceptives, progestogens, or oral estrogens, but these findings should be interpreted cautiously due to small subgroup sizes [11].

### 4.3. Outcomes in Patients with Repeated Implantation Failure

Some studies suggest that ES may particularly benefit patients with RIF [7,8,10], while others report limited effects [12,13]. Infertility in this group is often linked to impaired endometrial receptivity, making ES a promising treatment. A subgroup intention-to-treat analysis of one study found a significant increase in clinical pregnancy rates after ES in women with three or more implantation failures (45.5% vs. 27.5%; RR = 1.66; CI 1.01–2.78, *p* = 0.046). The per-protocol analysis also showed a significantly higher CPR (53.6% vs. 31.1%; RR = 1.72, CI 1.05–2.83, *p* = 0.024), OPR (46.4% vs. 26.7%; RR = 1.74, CI 1.00–3.05, *p* = 0.042), and LBR (46.4% vs. 26.7%; RR = 1.74, CI 1.00–3.05, *p* = 0.042) [8]. However, these results should be interpreted with caution, as the study did not account for a dropout rate exceeding 10% [8]. One study found ES to be an independent predictor of live births in women with two or more failed IVF cycles, using a double ES protocol in the luteal phase before treatment [7], whereas most other studies performed ES only once [8,9,10,11,12,13,14]. While some individual studies suggest improved outcomes in patients with RIF, meta-analyses provide mixed results. An individual patient data meta-analysis found no significant interaction between ES and factors such as the number of failed embryo transfers, age, infertility cause, or treatment type—although greater effects were observed in individual studies with a younger average age [17]. The van Hoogenhuijze et al., 2019 meta-analysis reported no significant effect of ES on the LBR or CPR in patients with one previous IVF failure [48]. Similarly, no significant improvement in the LBR was observed in patients undergoing their first IVF cycle or those with more than one implantation failure [48]. In contrast, the present analysis demonstrates significant improvements in both the CPR and LBR with negligible heterogeneity in patients with RIF. Other meta-analyses report higher ES efficacy in patients with RIF [16,18], with benefits increasing alongside the number of prior IVF failures [16].

### 4.4. Adverse Events

The adverse events reported in the included RCTs are provided in Supplementary Table S4. Across trials, any post-procedure symptoms occurred in 27.5–51.8% of women—most commonly, blood loss (6.3–43.0%) and abdominal pain (7.1–32.1%). Fever was rare (≈0–0.6%), and no serious complications or hospitalizations were reported [11,12]. Most patients (99.8%) found the procedure well tolerated, with median pain scores (VAS) of 4.0 at 30 min, 1.0 at 24 h, and 0.0 at day 7 post-procedure [11]. Studies report no increased risks of ectopic pregnancy, cesarean delivery, or adverse prenatal outcomes (e.g., preeclampsia, intrauterine growth restriction, gestational diabetes, preterm birth) [8,9,11]. Birth outcomes, including sex, birth weight, fetal malformations, and placental abnormalities, showed no significant differences [8]. Additionally, one study found lower rates of low birth weight, very low birth weight, and small for gestational age in the ES group [11]. No increased risk of miscarriage has been observed in any study [7,8,9], and no significant differences in multiple pregnancy rates were found [7,11].

### 4.5. Limitations

One limitation of this meta-analysis is the lack of subgroup analyses for RIF, infertility etiologies, or ovarian stimulation methods due to insufficient reported data. Stratified analyses separating these groups could provide clearer insights into ES efficacy and help to identify which patient populations may benefit most. Moreover, this meta-analysis was based solely on data reported in published studies; no authors were contacted for additional individual patient data. Access to individual patient data would have allowed for an analysis of factors influencing implantation success and outcomes following the ES procedure. Furthermore, variability in outcome definitions may have influenced the results. Some studies defined clinical pregnancy as the presence of a gestational sac with a detectable fetal heartbeat on ultrasound 4 weeks after embryo transfer [7], while others extended the period to 6 [9,12] or 8 weeks [11] or did not specify the timing of ultrasound evaluation [8,13]. Definitions of the OPR also varied. Some studies defined it as a detectable heartbeat at 10 weeks [12] or continuation beyond 12 weeks [9], while one study considered it the presence of at least one fetus with a heartbeat beyond 32 weeks [13]. Another study did not provide a specific definition [8]. Differences in outcomes across studies may have been influenced by the type of embryo used (fresh or frozen) and whether the embryos were autologous or donor-derived. One RCT used frozen embryos [13]; one used fresh or frozen embryos [11], while four used fresh embryos [7,8,9,12]. Five RCTs employed autologous embryos [7,8,11,12,13], and one used donor-derived embryos [9]. Additionally, variations in ovarian stimulation protocols across the included studies may have influenced the outcomes. Although similar meta-analyses exist [17], there remains a need for further investigations into the efficacy of ES from varying methodological perspectives. An individual participant data meta-analysis by van Hoogenhuijze et al. [17] did not restrict the timing of the ES procedure to a specific phase of the menstrual cycle, whereas our review focuses exclusively on studies in which ES was performed during the luteal phase. Our focus on the luteal phase is supported by evidence indicating that ES timing is critical: luteal-phase injury appears most favorable for upregulating implantation-related gene expression [7]. Furthermore, while their analysis considered the LBR as the sole primary outcome, our review includes the CPR and OPR as additional primary outcomes and reports corresponding RR and NNT estimates for each.

### 4.6. Implications

Future studies should aim to standardize the timing of ES—preferably in the luteal phase of the cycle preceding embryo transfer—and evaluate the impacts of variables such as the ES regimen (single vs. double), ovarian stimulation protocols, and embryo type on treatment outcomes. Further investigation into potential confounding factors, including age, parity, BMI, and RIF status, is warranted to identify patient subgroups that may derive the greatest benefit and to establish clear clinical indications for ES.

### 4.7. Summary of Findings

In patients undergoing IVF or ICSI, ES is associated with a 12% increase in LBR (RR = 1.12, 95% CI 1.03–1.22; high-certainty evidence; NNT = 22). The OPR is likely increased by 13% (RR = 1.13, 95% CI 1.01–1.26; moderate-certainty evidence; NNT = 20), and the CPR is improved by 9% (RR = 1.09, 95% CI 1.01–1.18; high-certainty evidence; NNT = 25). Between-study heterogeneity was negligible for all outcomes, indicating consistent effects. These findings suggest that ES may represent a clinically relevant intervention in ART.

## 5. Conclusions

Our meta-analysis shows that endometrial scratching has a small but statistically significant and consistent positive effect on clinical pregnancy, live birth, and ongoing pregnancy in women undergoing in vitro fertilization or intracytoplasmic sperm injection, corresponding to a modest absolute benefit of approximately 4–5 additional events per 100 individuals. The procedure is generally safe, with mild and transient adverse events, and does not appear to influence adverse prenatal or birth outcomes. The effectiveness of endometrial scratching in unselected patients remains uncertain. However, emerging evidence suggests that it may benefit women with repeated implantation failure, likely due to compromised endometrial receptivity, highlighting the need for further research in this specific subgroup.

## Figures and Tables

**Table 1:** Characteristics of included RCTs.

First Author, Year	Country	Recruitment Period	Type of Treatment	Study Population	Type of Embryo Transferred	Comparator	*N*	Mean Female Age in Years ± SD (Range)	Mean Female BMI in kg/m^2^ ± SD (Range)	Mean Duration of Infertility in Months ± SD (Range)	Risk of Bias
**van Hoogenhuijze, 2020** [12]	The Netherlands	January 2016 to July 2018	IVF/ICSI	Undergoing IVF/ICSI; at least one previous failed IVF/ICSI treatment	Fresh embryo	No intervention	933	ES group: 35.5 (31.8–39.0) Control group: 35.4 (31.4–38.7)	ES group: 23.5 (21.1–26.1) Control group: 24.1 (21.5–27.3)	ES group: 29.0 (20.0–43.0) Control group: 32.0 (20.0–45.0)	Low
**Olesen, 2019** [8]	Denmark	February 2014 to December 2017	IVF/ICSI	Undergoing IVF/ICSI; at least one previous failed IVF/ICSI treatment	Fresh embryo	No intervention	304	ES group: 31.9 ± 4.5 Control group: 31.9 ± 4.6	ES group: 23.8 ± 3.7 Control group: 24.1 ± 3.9	NI	Low
**Rodriguez, 2020** [9]	Spain	January 2017 to October 2018	IVF	Undergoing egg donor IVF	Fresh embryo, donor eggs	No intervention	352	ES group: 41.29 ± 4.22 Control group: 41.50 ± 4.63	ES group: 22.78 ± 3.39 Control group: 22.92 ± 3.31	NI	Low
**Gibreel, 2015** [7]	Egypt	NI	IVF	Undergoing IVF; at least one previous failed IVF treatment	Fresh embryo	Endocervical manipulation	387	ES group: 30.2 ± 4.2 Control group: 30.6 ± 3.9	ES group: 26.2 ± 2.5 Control group: 26.3 ± 2.6	ES group: 96 ± 40.8 Control group: 100.8 ± 48	Low
**Metwally, 2021** [11]	United Kingdom	July 2016 to October 2019	IVF/ICSI	Undergoing first IVF/ICSI cycle	Fresh or frozen embryo	No intervention	1048	ES group: 32.7 ± 3.3 (21.4–38.1) Control group: 32.4 ± 3.4 (21.4–38.8)	ES group: 24.5 ± 3.3 (16.8, 34.9) Control group: 24.5 ± 3.4 (17.3–35)	ES group: 37.2 ± 22.8 (0–216) Control group: 37.2 ± 20.4 (0–180)	Some concerns
**Nastri, 2013** [10]	Brazil	June 2010 to March 2012	IVF/ICSI	Undergoing IVF	Fresh embryo	Drying of the cervix	158	ES group: 32.4 ± 3.2 Control group: 32.1 ± 3.1	NI	NI	High
**Mak, 2017** [13]	China	March 2013 to April 2016	IVF/ICSI	Undergoing IVF/ICSI, at least one previous failed IVF treatment	Frozen embryo	Endocervical manipulation	Per protocol—186	ES group: 36.90 ± 3.32 Control group: 36.78 ± 3.45	ES group: 21.36 ± 2.81 Control group: 21.90 ± 2.81	ES group: 56.88 ± 43.44 Control group: 56.76 ± 43.44	Some concerns
**Frantz, 2019** [14]	France	February 2010 to July 2014	IVF/ICSI	Undergoing first or second IVF/ICSI attempt	Fresh embryo	No intervention	As treated—132	ES group: 32.8 ± 4.0 Control group: 32.9 ± 3.4	ES group: 23.2 ± 4.3 Control group: 22.4 ± 3.0	ES group: 41.7 ± 22.6 Control group: 49.3 ± 26.9	High

NI—no information; IVF—in vitro fertilization; ICSI—intracytoplasmic sperm injection; ES—endometrial scratching; *N*—number of participants; SD—standard deviation; BMI—body mass index.

**Table 2:** Pooled baseline characteristics of participants.

	Main Analysis (6 Trials)		All Trials (8 Trials)	
	Endometrial Scratching (*N* = 1603)	Control (*N* = 1607)	Endometrial Scratching (*N* = 1750)	Control (*N* = 1750)
**Mean female age, years (±SD)**	34.89 (±5.33)	34.96 (±5.37)	34.76 (±5.26)	34.85 (±5.29)
**Mean female BMI, kg/m^2^ (±SD) ^a^**	23.99 (±3.01)	24.28 (±3.05)	23.96 (±3.07)	24.21 (±3.07)
**Mean duration of infertility, months (±SD) ^b^**	44.53 (±33.68)	46.38 (±35.17)	44.38 (±33.21)	46.52 (±34.82)
**Smoking status, *N* (%) ^c^**				
Yes	102 (8.7)	104 (8.9)	121 (9.8)	111 (9.0)
No	1064 (91.3)	1063 (91.1)	1113 (90.2)	1120 (91.0)
**Number of previous failed ETs, *N* (%) ^d^**				
0	523 (39.2)	525 (39.2)	531 (37.6)	535 (37.8)
1	643 (48.2)	651 (48.7)	670 (47.4)	673 (47.5)
2 and more	168 (12.6)	162 (12.1)	212 (15.0)	209 (14.7)
**Cause of infertility, *N* (%) ^e^**				
Idiopathic/unexplained	188 (11.7)	192 (11.9)	203 (11.6)	202 (11.54)
Male factor	350 (21.8)	325 (20.2)	384 (21.94)	357 (20.4)
Tubal factor	77 (4.8)	80 (5.0)	77 (4.4)	80 (4.57)
Ovulatory	18 (1.1)	18 (1.1)	18 (1.03)	16 (0.91)
Endometriosis	23 (1.4)	27 (1.7)	23 (1.31)	27 (1.54)
Other	18 (1.1)	25 (1.6)	18 (1.03)	25 (1.43)
Mixed	52 (3.2)	66 (4.1)	56 (3.2)	78 (4.46)
Not specified	892 (55.6)	899 (55.9)	971 (55.49)	974 (55.66)
**Type of infertility, *N* (%) ^f^**				
Primary	291 (18.2)	288 (17.9)	347 (19.8)	339 (19.4)
Secondary	269 (16.8)	271 (16.9)	281 (16.1)	284 (16.2)
Not reported	1043 (65.1)	1048 (65.2)	1122 (64.1)	1127 (64.4)

^a^ Mean BMI was not reported in the study by Nastri et al. [10]; therefore, it was excluded from the pooled analyses of the mean BMI. ^b^ Mean duration of infertility was not reported in the studies by Olesen et al. [8], Rodriguez et al. [9], and Nastri et al. [10], and these trials were excluded from the pooled analyses of the infertility duration. ^c^ Smoking status was not reported by Olesen et al. [8], Gibreel et al. [7], Nastri et al. [10], or Mak et al. [13]. These trials were excluded from the pooled smoking analyses. ^d^ The number of previous failed IVF treatments was heterogeneously reported across trials. Data were harmonized into three categories (0, 1, ≥2 previous failed IVF). Studies that did not report categorical data (Rodriguez et al. [9], Mak et al. [13]) or reported non-separable categories (Frantz et al. [14], reporting “≥1”) were excluded. ^e^ Some patients presented with more than 1 cause of infertility. The cause of infertility was not reported in the studies by Rodriguez et al. [9], Gibreel et al. [7], Metwally et al. [11], and Nastri et al. [10] and therefore could not be included in the pooled analyses. ^f^ Primary: female has never conceived before. Secondary: female has conceived before. Data missing for 599 patients in the ES group and 602 in the control group. Abbreviations: BMI- body mass index; SD-standard deviation; ET- embryo transfer.

**Table 3:** Summary outcome statistics.

Study	CPR				LBR				OPR				Risk of Bias Assessment
	ES Group *n*/*N* (%)	Control Group *n*/*N* (%)	RR (CI 95%)	NNT	ES Group *n*/*N* (%)	Control Group *n*/*N* (%)	RR (CI 95%)	NNT	ES Group *n*/*N* (%)	Control Group *n*/*N* (%)	RR (CI 95%)	NNT	
van Hoogenhuijze, 2020 [12]	234/467 (50.1)	211/466 (45.3)	1.11 (0.97; 1.27)	20.7	202/467 (43.3)	178/466 (38.2)	1.13 (0.97; 1.32)	19.8	208/467 (44.5)	186/466 (39.9)	1.12 (0.96; 1.30)	21.6	Low
Olesen, 2019 [8]	55/151 (36.4)	50/153 (32.7)	1.11 (0.82; 1.52)	26.7	47/151 (31.1)	37/153 (24.2)	1.29 (0.89; 1.86)	14.4	47/151 (31.1)	37/153 (24.2)	1.29 (0.89; 1.86)	14.4	Low
Izquierdo Rodriguez, 2020 [9]	108/176 (61.4)	102/176 (58.0)	1.06 (0.89; 1.26)	29.3	96/176 (54.5)	85/176 (48.3)	1.13 (0.92; 1.39)	16	97/176 (55.1)	87/176 (49.4)	1.11 (0.91; 1.37)	17.6	Low
Gibreel et al., 2015 [7]	95/193 (49.2)	80/194 (41.2)	1.24 (0.98; 1.56)	12.5	91/193 (47.2)	74/194 (38.1)	1.19 (0.96; 1.49)	11.1	-	-	-	-	Low
Metwally, 2021 [11]	223/523 (42.6)	213/525 (40.6)	1.05 (0.91; 1.21)	48.4	202/523 (38.6)	195/525 (37.1)	1.04 (0.89; 1.21)	67.5	-	-	-	-	Some concerns
Nastri, 2013 [10]	39/79 (49.37)	23/79 (29.11)	1.70 (1.13; 2.56) ^a^	4.9	33/79 (41.77)	18/79 (22.78)	1.83 (1.13–2.97) ^a^	5.3	-	-	-	-	High
Frantz, 2019 [14]	16/64 (23.5)	23/68 (35.9)	0.65 (0.38; 1.12)	8.1	-	-	-	-	14/64 (20.6)	20/68 (31.7)	0.66 (0.36; 1.19)	9.4	High
Mak, 2017 [13]	39/93 (41.9)	35/93 (37.6)	1.114 (0.782; 1.588)	23.3	32/93 (34.4)	29/93 (31.2)	1.158 (0.627; 2.137)	31	32/93 (34.4)	29/93 (31.2)	1.158 (0.627; 2.137)	31	Some concerns

CPR—clinical pregnancy rate; LBR—live birth rate; OPR—ongoing pregnancy rate; RR—relative risk; CI—confidence interval; ES—endometrial scratching; NNT—number needed to treat. ^a^—statistically significant value.

## Data Availability

The original contributions presented in this study are included in the article. Further inquiries can be directed to the corresponding author.

## Supplementary Materials

The following supporting information can be downloaded at https://www.mdpi.com/article/10.3390/jcm15093340/s1. Figure S1: Risk of bias assessment; Figure S2: Certainty evaluation; Table S1: Definitions of outcomes in included studies; Table S2: Endometrial scratching techniques, devices, operator variability; Table S3: Ovarian stimulation and luteal-phase support protocols in included trials; Table S4: Adverse events after endometrial scratching; Table S5: PRISMA checklist.

## Abbreviations

The following abbreviations are used in this manuscript:

ARD	Absolute risk difference
CPR	Clinical pregnancy rate
CI	Confidence interval
ES	Endometrial scratching
NNT	Number needed to treat
ICSI	Intracytoplasmic sperm injection
IVF	In vitro fertilization
LBR	Live birth rate
OPR	Ongoing pregnancy rate
RIF	Repeated implantation failure
RR	Risk ratio
