# Supplementary material for: Impact of Endometrial Scratching on IVF/ICSI Outcomes: A Meta-Analysis
Source: J Clin Med. 2026 Apr 27;15(9):3340. doi: 10.3390/jcm15093340 (PMC13163817; doi:10.3390/jcm15093340)
Supplement: Supplementary file 1 [file jcm-15-03340-s001.zip › jcm-4186516-supplementary.pdf]

Figure S1. Risk of bias assessment.

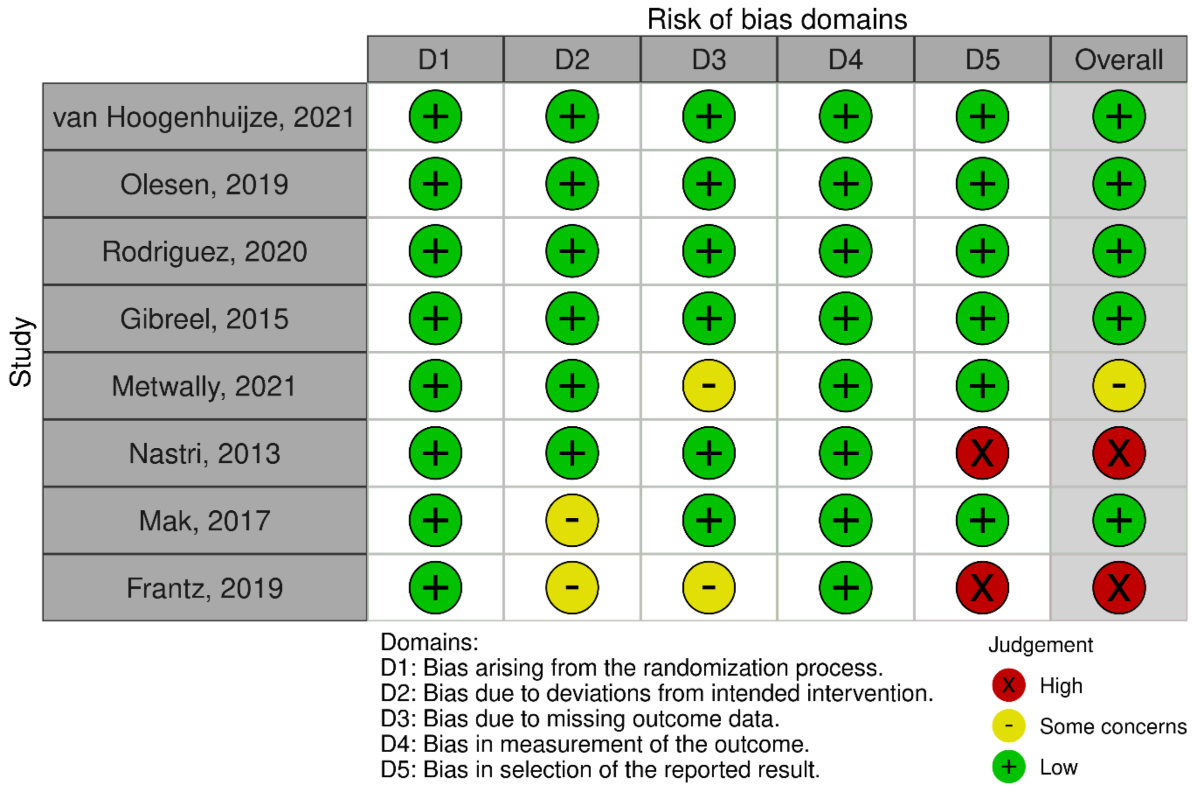

Figure S2. Certainty evaluation.

Certainty assessment							№ of patients		Effect		Certainty	Importance
№ of studies	Study design	Risk of bias	Inconsistency	Indirectness	Imprecision	Other considerations	endometrial scratching	control	Relative (95% CI)	Absolute (95% CI)		
**Live Birth Rate**												
6	randomised trials	not serious	not serious	not serious	not serious	none	674/1278 (52.7%)	604/1278 (47.3%)	**RR 1.12** (1.03 to 1.22)	**57 more per 1,000** (from 14 more to 104 more)	⨁⨁⨁⨁ High	IMPORTANT
**Ongoing Pregnancy Rate**												
4	randomised trials	not serious	not serious	serious[a]	not serious	none	384/723 (53.1%)	339/723 (46.9%)	**RR 1.13** (1.01 to 1.26)	**61 more per 1,000** (from 5 more to 122 more)	⨁⨁⨁◯ Moderate[a]	IMPORTANT
**Clinical Pregnancy Rate**												
6	randomised trials	not serious	not serious	not serious	not serious	none	750/1435 (52.3%)	685/1435 (47.7%)	**RR 1.09** (1.01 to 1.18)	**43 more per 1,000** (from 5 more to 86 more)	⨁⨁⨁⨁ High	IMPORTANT

CI: confidence interval; **RR:** risk ratio

Explanations

a. variations in definitions

Table S1. Definitions of outcomes in included studies.

Author, year	Clinical pregnancy rate	Live birth rate	Ongoing pregnancy rate
**N E van Hoogenhuijze, 2021**	Rate of pregnancies with an intrauterine gestational sac visible on ultrasound at 6–7 weeks.	Rate of ongoing pregnancies resulting in live birth within 12 months of randomisation.	Rate of pregnancies continuing beyond 10 weeks of gestation.
**Mia Steengaard Olesen, 2019**	Definition not provided	Definition not provided	Definition not provided
**Izquierdo Rodriguez, 2020**	Rate of pregnancies with an intrauterine gestational sac confirmed by ultrasound at ~6 weeks.	Rate of live births beyond 24 weeks of pregnancy.	Rate of pregnancies continuing beyond 12 weeks of gestation.
**Gibreel et al., 2015**	Rate of pregnancies with confirmed intrauterine gestational sac and fetal heartbeat at 4 weeks.	Rate of live births beyond 24 weeks of pregnancy.	-
**Metwally M, 2021**	Rate of viable intrauterine pregnancies with heartbeat seen on ultrasound at/after 8 weeks.	Rate of live births beyond 24 weeks of pregnancy.	-
**Nastri CO, 2013**	Rate of pregnancies with at least one fetus showing a heartbeat.	Rate of deliveries with at least one live-born infant.	-
**Mak JSM, 2017**	Rate of pregnancies with confirmed intrauterine gestational sac.	Rate of deliveries with at least one live-born infant.	Rate of pregnancies continuing beyond 32 weeks of gestation.
**Frantz S, 2019**	Rate of pregnancies with confirmed intrauterine gestational sac and fetal heartbeat.	-	Rate of pregnancies that continued beyond 12 weeks.

Supplementary Table S2. Endometrial scratching techniques, devices, operator variability.

Article	Technique	Devices	Operator
N E van Hoogenhuijze, 2021	Suction and rotation	Endometrial biopsy catheter, not specified	3 operators, not detailed
Mia Steengaard Olesen, 2019	Not detailed	Pipelle de Cornier	Not detailed
Izquierdo Rodriguez, 2020	Suction	Pipelle de Cornier	Not detailed
Gibreel et al., 2015	Back-and-forth passes	Pipelle de Cornier	Not detailed
Metwally M, 2021	Back-and-forth passes	Pipelle sampler or similar device	Not detailed
Nastri CO, 2013	Back-and-forth passes	Pipelle de Cornier	One physician
Mak JSM, 2017	Suction	Pipette	Not detailed
Frantz S, 2019	Suction and rotation	Pipelle de Cornier	Not detailed

Table S3. Ovarian Stimulation and Luteal-Phase Support Protocols in Included Trials

Study	Cycle Type	Ovarian Stimulation Protocol	Luteal-Phase Support
Frantz S, 2019	Fresh IVF/ICSI	Conventional gonadotropin stimulation; long GnRH-agonist or GnRH-antagonist protocol.	Not described; standard progesterone luteal support presumed.
Gibreel et al., 2015	Fresh IVF/ICSI	COCP pretreatment → long GnRH-agonist down-regulation → gonadotropins.	Micronized progesterone twice daily; until negative test or 12 weeks.
Nastri CO, 2013	Fresh IVF/ICSI	Three regimens: (1) CC + hMG + antagonist; (2) rFSH + antagonist; (3) rFSH + long agonist.	Micronized progesterone 600 mg/day; to β-hCG or 12 weeks.
Mia Steengaard Olesen, 2019	Fresh IVF/ICSI	Standardized rFSH + antagonist; antagonist from day 5; hCG trigger.	Vaginal progesterone (Crinone 90 mg daily) until pregnancy test.
Metwally M, 2021	Fresh IVF/ICSI	Pragmatic; routine stimulation per clinic (agonist or antagonist).	Routine progesterone luteal support (not detailed).
N E van Hoogenhuijze, 2021	Fresh IVF/ICSI	Pragmatic; clinic-standard stimulation (agonist or antagonist).	Routine progesterone luteal support (not detailed).
Mak JSM, 2017	Natural-cycle FET	No stimulation; natural cycle ovulation monitoring.	Luteal support not routinely given.
Izquierdo Rodriguez, 2020	Donor-egg recipient	Recipients: estradiol-only endometrial preparation. Donors stimulated but not clinically relevant.	Vaginal micronized progesterone 400 mg q12h until pregnancy test.

* IVF - In vitro fertilization; ICSI - Intracytoplasmic sperm injection; COS - Controlled ovarian stimulation; GnRH - Gonadotropin-releasing hormone; GnRH agonist (long protocol) - Down-regulation protocol using a GnRH agonist; GnRH antagonist protocol - Protocol preventing premature LH surge; rFSH - Recombinant follicle-stimulating hormone; hMG - Human menopausal gonadotropin; CC - Clomiphene citrate; COCP - Combined oral contraceptive pill; hCG - Human chorionic gonadotropin; LH - Luteinizing hormone; FET - Frozen embryo transfer; HRT cycle - Hormone replacement therapy cycle; q12h - Every 12 hours; β-hCG - Beta–human chorionic gonadotropin.

Table S4. Adverse events after endometrial scratching.

Study	N	Any, % (n)	Abdominal pain, % (n)	Blood loss, % (n)	Fever, % (n)	Vaginal discharge, % (n)
N E van Hoogenhuijze, 2021	467	51.82% (242)	32.12% (150)	43.04% (201)	0.64% (3)	
Mia Steengaard Olesen, 2019*	151					
Izquierdo Rodriguez, 2020	176	Not reported				
Gibreel et al., 2015	193	Not reported				
Metwally M, 2021	523	27.53% (144)	7.07% (37)	6.31% (33)		
Mak JSM, 2017	93	Not reported				

* No uterine infections, bleeding, or adverse events reported, besides a short pain during the endometrial scratching procedure.

Table S5. PRISMA checklist.

Section and Topic	Item #	Checklist item	Location where item is reported
**TITLE**			
Title	1	Identify the report as a systematic review.	P1, title
**ABSTRACT**			
Abstract	2	See the PRISMA 2020 for Abstracts checklist.	+
**INTRODUCTION**			
Rationale	3	Describe the rationale for the review in the context of existing knowledge.	1.Introduction.
Objectives	4	Provide an explicit statement of the objective(s) or question(s) the review addresses.	1.Introduction, last sentence.
**METHODS**			
Eligibility criteria	5	Specify the inclusion and exclusion criteria for the review and how studies were grouped for the syntheses.	2.1 Search strategy and study selection.
Information sources	6	Specify all databases, registers, websites, organisations, reference lists and other sources searched or consulted to identify studies. Specify the date when each source was last searched or consulted.	2.1 Search strategy and study selection.
Search strategy	7	Present the full search strategies for all databases, registers and websites, including any filters and limits used.	2.1 Search strategy and study selection.
Selection process	8	Specify the methods used to decide whether a study met the inclusion criteria of the review, including how many reviewers screened each record and each report retrieved, whether they worked independently, and if applicable, details of automation tools used in the process.	2.1. Search strategy and study selection
Data collection process	9	Specify the methods used to collect data from reports, including how many reviewers collected data from each report, whether they worked independently, any processes for obtaining or confirming data from study investigators, and if applicable, details of automation tools used in the process.	2.3. Data extraction and statistical analysis.
Data items	10a	List and define all outcomes for which data were sought. Specify whether all results that were compatible with each outcome domain in each study were sought (e.g. for all measures, time points, analyses), and if not, the methods used to decide which results to collect.	2.3. Data extraction and statistical analysis.
	10b	List and define all other variables for which data were sought (e.g. participant and intervention characteristics, funding sources). Describe any assumptions made about any missing or unclear information.	2.3. Data extraction and statistical analysis.<br>2.4. Missing data
Study risk of bias assessment	11	Specify the methods used to assess risk of bias in the included studies, including details of the tool(s) used, how many reviewers assessed each study and whether they worked independently, and if applicable, details of automation tools used in the process.	2.2. Risk of bias assessment.
Effect measures	12	Specify for each outcome the effect measure(s) (e.g. risk ratio, mean difference) used in the synthesis or presentation of results.	2.3. Data extraction and statistical analysis.
Synthesis methods	13a	Describe the processes used to decide which studies were eligible for each synthesis (e.g. tabulating	2.1. Search strategy and study selection.

Section and Topic	Item #	Checklist item	Location where item is reported
		the study intervention characteristics and comparing against the planned groups for each synthesis (item #5)).	
	13b	Describe any methods required to prepare the data for presentation or synthesis, such as handling of missing summary statistics, or data conversions.	2.3. Data extraction and statistical analysis.<br>2.4. Missing data.
	13c	Describe any methods used to tabulate or visually display results of individual studies and syntheses.	2.3. Data extraction and statistical analysis.
	13d	Describe any methods used to synthesize results and provide a rationale for the choice(s). If meta-analysis was performed, describe the model(s), method(s) to identify the presence and extent of statistical heterogeneity, and software package(s) used.	2.3. Data extraction and statistical analysis.
	13e	Describe any methods used to explore possible causes of heterogeneity among study results (e.g. subgroup analysis, meta-regression).	2.3. Data extraction and statistical analysis.
	13f	Describe any sensitivity analyses conducted to assess robustness of the synthesized results.	2.2. Risk of bias assessment.<br>2.3. Data extraction and statistical analysis.
Reporting bias assessment	14	Describe any methods used to assess risk of bias due to missing results in a synthesis (arising from reporting biases).	2.2. Risk of bias assessment
Certainty assessment	15	Describe any methods used to assess certainty (or confidence) in the body of evidence for an outcome.	2.2. Risk of bias assessment.
**RESULTS**			
Study selection	16a	Describe the results of the search and selection process, from the number of records identified in the search to the number of studies included in the review, ideally using a flow diagram.	2.1. Search strategy and study selection. Figure 1.
	16b	Cite studies that might appear to meet the inclusion criteria, but which were excluded, and explain why they were excluded.	2.1. Search strategy and study selection.
Study characteristics	17	Cite each included study and present its characteristics.	2.1. Search strategy and study selection. Table 1.
Risk of bias in studies	18	Present assessments of risk of bias for each included study.	2.2. Risk of bias assessment.
Results of individual studies	19	For all outcomes, present, for each study: (a) summary statistics for each group (where appropriate) and (b) an effect estimate and its precision (e.g. confidence/credible interval), ideally using structured tables or plots.	3.3 Clinical pregnancy rate, live birth rate, ongoing pregnancy rate, Table 3
Results of syntheses	20a	For each synthesis, briefly summarise the characteristics and risk of bias among contributing studies.	Table 1. Table 3.
	20b	Present results of all statistical syntheses conducted. If meta-analysis was done, present for each the summary estimate and its precision (e.g. confidence/credible interval) and measures of statistical heterogeneity. If comparing groups, describe the direction of the effect.	3.3 Clinical pregnancy rate, live birth rate, ongoing pregnancy rate. Table 3. Figure 2.
	20c	Present results of all investigations of possible causes of heterogeneity among study results.	3.3 Clinical pregnancy rate, live birth rate, ongoing pregnancy rate.
	20d	Present results of all sensitivity analyses conducted to assess the robustness of the synthesized results.	3.3 Clinical pregnancy rate, live birth rate, ongoing pregnancy rate.

Section and Topic	Item #	Checklist item	Location where item is reported
Reporting biases	21	Present assessments of risk of bias due to missing results (arising from reporting biases) for each synthesis assessed.	2.2. Risk of bias assessment. 2.4. Missing data
Certainty of evidence	22	Present assessments of certainty (or confidence) in the body of evidence for each outcome assessed.	2.2. Risk of bias assessment.<br>3.3 Clinical pregnancy rate, live birth rate, ongoing pregnancy rate. Table 3. Figure 2.
**DISCUSSION**			
Discussion	23a	Provide a general interpretation of the results in the context of other evidence.	Discussion: 4.2. Outcomes in unselected patients; 4.3. Outcomes in patients with repeated implantation failures.
	23b	Discuss any limitations of the evidence included in the review.	4.5. Limitations
	23c	Discuss any limitations of the review processes used.	4.5. Limitations
	23d	Discuss implications of the results for practice, policy, and future research.	4.6. Implications
**OTHER INFORMATION- PROSPERO**			
Registration and protocol	24a	Provide registration information for the review, including register name and registration number, or state that the review was not registered.	P1, Trial registration. 2.1. Search strategy and study selection.
	24b	Indicate where the review protocol can be accessed, or state that a protocol was not prepared.	2.1. Search strategy and study selection.
	24c	Describe and explain any amendments to information provided at registration or in the protocol.	Not applicable
Support	25	Describe sources of financial or non-financial support for the review, and the role of the funders or sponsors in the review.	P1, Funding statement
Competing interests	26	Declare any competing interests of review authors.	P1, Disclosure statement
Availability of data, code and other materials	27	Report which of the following are publicly available and where they can be found: template data collection forms; data extracted from included studies; data used for all analyses; analytic code; any other materials used in the review.	P1, Attestation statements

From: Page MJ, McKenzie JE, Bossuyt PM, Boutron I, Hoffmann TC, Mulrow CD, et al. The PRISMA 2020 statement: an updated guideline for reporting systematic reviews. BMJ 2021;372:n71. doi: 10.1136/bmj.n71. This work is licensed under CC BY 4.0. To view a copy of this license, visit https://creativecommons.org/licenses/by/4.0/
